# Vitamin D deficiency and inflammatory markers in type 2 diabetes: Big data insights

**DOI:** 10.1515/biol-2022-0787

**Published:** 2024-05-28

**Authors:** Rujie Shan, Qi Zhang, Yusen Ding, Lei Zhang, Yanhu Dong, Weiguo Gao

**Affiliations:** Weifang Medical University, Clinical Medical, Weifang, 261000, China; Qingdao Endocrine Diabetes Hospital, Qingdao, 266000, China

**Keywords:** type 2 diabetes mellitus, 25-hydroxyvitamin D, markers of inflammation

## Abstract

The objective of this study was to investigate the prevalence of vitamin D deficiency or insufficiency and its association with inflammatory markers and type 2 diabetes. We conducted our research at Qingdao Endocrine and Diabetes Hospital, where serum 25-hydroxyvitamin D3 levels were determined for 2,806 individuals with type 2 diabetes aged 30 and older between January 2018 and December 2019. Medical records were analyzed, and data on health, blood lipids, HbA1c, and inflammation were collected. Our results revealed a high prevalence of vitamin D deficiency in the population. Among male and female patients, median serum 25(OH)D3 levels were 22.46 and 19.00 ng/mL, respectively. More than 60% of female patients had vitamin D deficiency, with over 80% having levels below 30 ng/mL. We observed a favorable connection between high-density lipoprotein cholesterol and 25(OH)D3, while triglycerides and HbA1c showed negative correlations. As 25(OH)D3 levels increased, inflammatory markers such as hypersensitive C-reactive protein (hsCRP), erythrocyte sedimentation rate (ESR), white blood cell count, neutrophil count, and monocyte count decreased (trend test, *p* < 0.05), although peripheral blood lymphocytes initially increased and then decreased. After controlling for age and gender, multiple linear regression analysis indicated negative correlations between ESR, hsCRP, and white blood cell count with 25(OH)D3 (*p* < 0.05). In conclusion, our study demonstrates that individuals with type 2 diabetes often exhibit vitamin D deficiency or insufficiency, which is associated with elevated levels of inflammatory markers in the blood.

## 1 Introduction

Diabetes is a chronic disease. It occurs when the pancreas does not produce enough insulin or the body is unable to use the insulin produced efficiently. Insulin is a hormone that regulates blood sugar. Hyperglycemia or elevated blood sugar is a common consequence of uncontrolled diabetes and can cause serious damage to many of the body’s systems, especially nerves and blood vessels, over time. Symptoms of diabetes can occur suddenly and can be mild and can take many years to notice. Symptoms of diabetes include feeling thirsty, needing to urinate more often than usual, blurred vision, feeling tired, and unintentional weight loss. Over time, diabetes can damage the heart, blood vessels, eyes, kidneys, and nerves. People with diabetes are at higher risk of developing health problems, including heart attack, stroke, and kidney failure. Diabetes damages the blood vessels in the eye, leading to permanent vision loss. Many people with diabetes have foot problems due to nerve damage and poor blood flow. This can lead to foot ulcers and can lead to amputation.

Type 2 diabetes affects the way the body uses sugar (glucose) for energy. It prevents the body from using insulin properly, which can cause blood sugar to rise if left untreated. Symptoms of type 2 diabetes may be mild, and may take years to notice. Symptoms may be similar to type 1 diabetes, but they are often not obvious. Therefore, diagnosis may not be made until several years after onset and complications. More than 95% of people with diabetes have type 2 diabetes. Type 2 diabetes was previously known as non-insulin-dependent diabetes or adult-onset diabetes. Until recently, this type of diabetes was only seen in adults, but it is now becoming more frequent in children.

Multiple studies have shown a close correlation between chronic low-grade inflammation and the occurrence and development of type 2 diabetes mellitus (T2DM) and its complications [1]. Even some scholars believe that T2DM is a chronic low-grade inflammatory disease [2]. A 2013 meta-analysis reported a significant dose–response association (RR = 1.31) between IL-6 levels and the risk of T2DM, and a significant correlation between elevated CRP levels and an increased risk of T2DM (RR = 1.26) [3]. A prospective study report in 2016 pointed out that hs CRP may be closely related to the occurrence and development of T2DM and its major vascular complications [4].

There are many causes of chronic mild inflammation, among which vitamin D deficiency/deficiency is increasingly being valued. In 2005, Mario Flores conducted a comprehensive analysis that reported a close association between [5] 1,25(OH)2D3 and low-intensity chronic inflammation in T2DM patients. In 2017, Jiang Ling et al. in China found through a case–control study that there was a negative correlation between 25(OH)D3 and the expression of IL-6, IL-10, and hs CRP [6]. In 2022, a bidirectional Mendelian randomization study conducted by the University of South Australia confirmed a direct relationship between low levels of vitamin D and high levels of inflammation, with CRP levels decreasing with an increase in 25 hydroxy vitamin D (25(OH)D). The above studies have analyzed the relationship between 25(OH)D and CRP, but the relationship with other inflammatory markers is not yet clear [7].

Therefore, this study aims to further analyze the correlation between vitamin D deficiency and inflammatory markers in the blood of T2DM patients.

## 2 Materials and methods

### 2.1 Study population

From January 2018 to December 2019, 2806 T2DM patients (1,501 males) aged 30 years and above who were hospitalized in Qingdao Endocrine Diabetes Hospital and whose serum 25(OH)D3 was measured for the first time were selected as the study subjects.


**Inclusion criteria:** (1) All the selected patients met the WHO diagnostic criteria for diabetes in 1999 and (2) had not taken any vitamin D preparations or drugs that affect vitamin D metabolism within the first 6 months of the group.
**Exclusion criteria:** (1) patients suffering from severe heart, lung, liver, kidney, and other important organ failures; (2) patients diagnosed with malignant tumors; (3) patients who had received organ transplant surgery and/or immunosuppressive therapy before; (4) patients who had chronic diarrhea or other diseases that clearly affect the absorption of vitamin D, and taking medication that affects the absorption of fat-soluble vitamins; and (5) patients with acute and chronic infections.

### 2.2 Data collection

All enrolled patients’ gender, age, occupation, height, weight, waist circumference, course of diabetes, clinical diagnosis and liver function, renal function, 25(OH)D3, HbA1C, hypersensitive C-reactive protein (hsCRP), erythrocyte sedimentation rate (ESR), peripheral blood routine, white blood cell count, neutrophil count (NEUT), lymphocyte count (LYMPH), monocyte count (MONO), and other data were collected.

### 2.3 Diagnostic criteria

#### 2.3.1 Diagnostic criteria for T2DM

The diagnostic criteria and typing criteria for diabetes of the World Health Organization (WHO) in 1999 were adopted [8]: fasting blood glucose (FPG) level ≥7.0 mmol/L and/or postprandial blood glucose level ≥11.1 mmol/L were the classification criteria.

#### 2.3.2 Diagnostic criteria for vitamin D

25(OH)D3 levels >30 ng/mL indicate sufficient vitamin D, 25(OH)D3 levels between 20 and 30 ng/mL indicate insufficient vitamin D, and 25(OH)D3 <20 ng/mL indicates vitamin D deficiency [9].

### 2.4 Statistical methods

Statistical analysis was conducted using SPSS27.0 software. The measurement data were consistent with a normal distribution, represented by mean ± standard deviation ( \[\bar{x}]\] + s). Analysis of variance and *t*-tests were used for inter-group comparisons. Non-parametric tests were used for non-normal distributions. Counting data were presented in frequency (*n*) and percentage (%). Chi square tests were used for inter-group comparisons, and multiple linear regression was used for correlation analysis. *p* < 0.05 indicates statistical significance.


**Informed consent:** Informed consent was obtained from all individuals included in this study.
**Ethical approval:** The research related to human use complied with all the relevant national regulations, institutional policies and is in accordance with the tenets of the Helsinki Declaration, and has been approved by the Medical Ethics Committee of our hospital.

## 3 Results

### 3.1 General clinical features

As shown in Table 1, there is no significant difference between the patients in 2018 and 2019 in average age, diabetes course, obesity degree, HbA1c, 25(OH)D3, ESR, hsCRP, etc., therefore, this study will combine the patient data of 2 years for analysis.

**Table 1:** Clinical characteristics and vitamin D levels of T2DM patients in different years ( \[\bar{x}\left+s)]\]

	2018	2019
**Male** (number)	683	818
Age (years)	57.94 ± 11.51	58.58 ± 10.67
BMI (kg/m^2^)	26.10 ± 3.31	26.20 ± 3.63
HBA1c (%)	8.36 ± 2.09	8.09 ± 2.02
ESR (mm/h)	12.76 ± 16.33	11.98 ± 14.09
hsCRP (mg/L)	8.04 ± 24.61	6.21 ± 19.14
TG (mmol/L)	2.03 ± 2.03	2.05 ± 2.28
TC (mmol/L)	5.56 ± 1.36	5.25 ± 1.33
HDL-C (mmol/L)	1.27 ± 0.34	1.20 ± 0.29
LDL-C (mmol/L)	2.90 ± 1.00	2.86 ± 0.94
25(OH)D insufficiency (%)	36.46	41.20
25(OH)D deficiency (%)	49.78	38.02
**Female** (number)	644	661
Age (years)	61.96 ± 10.88	62.16 ± 10.62
BMI (kg/m^2^)	25.61 ± 3.72	25.53 ± 3.54
HBA1c (%)	8.32 ± 2.12	8.01 ± 2.01
ESR (mm/h)	20.40 ± 17.29	20.43 ± 16.83
hsCRP (mg/L)	6.00 ± 18.79	6.36 ± 17.57
TG (mmol/L)	1.87 ± 1.87	1.82 ± 1.35
TC (mmol/L)	6.08 ± 1.39	5.63 ± 1.25
HDL-C (mmol/L)	1.41 ± 0.31	1.35 ± 0.32
LDL-C (mmol/L)	3.22 ± 1.03	3.10 ± 0.97
25(OH)D insufficiency (%)	26.40	31.77
25(OH)D deficiency (%)	67.08	58.40

### 3.2 Vitamin D levels

As shown in Table 1, vitamin D insufficiency and deficiency are commonly present in this group of T2DM patients. The average levels of blood 25(OH)D3 in male and female patients were (22.46 ± 9.53) ng/mL and (19.00 ± 8.51) ng/mL, respectively. More than 80% of patients have 25(OH)D levels below 30 ng/mL. The situation of low 25(OH)D3 levels in female is particularly significant, with a prevalence of vitamin D deficiency exceeding 60%.

### 3.3 Metabolic disorders in patients with 25(OH)D3 and T2DM

As shown in Table 2, with the increase of vitamin D levels, the average levels of HbA1c and triglycerides (TG) decrease, while the average levels of high-density lipoprotein cholesterol (HDL-C) increase, indicating a positive trend test (*p* < 0.05).

**Table 2:** Glucose and lipid metabolism disorders in patients with different 25(OH)D3 levels

	0	1	2	3	4	5	6	7	8	9	*p*
**Male**											
BMI (kg/㎡)	25.46 ± 5.71	26.64 ± 3.88	26.40 ± 3.41	25.90 ± 3.31	26.28 ± 3.64	26.25 ± 3.40	26.71 ± 4.02	25.93 ± 3.19	26.22 ± 3.31	25.82 ± 3.17	0.110
HBA1c (%)	9.18 ± 2.32	8.51 ± 2.20	8.90 ± 2.10	8.22 ± 1.96	8.46 ± 2.05	8.34 ± 1.89	8.12 ± 2.04	8.09 ± 2.06	7.69 ± 1.69	7.62 ± 1.98	<0.001
TG (mmol/L)	2.42 ± 3.37	2.32 ± 2,78	2.68 ± 3.09	2.09 ± 1.78	2.17 ± 2.00	2.13 ± 1.81	1.76 ± 1.48	1.95 ± 1.90	1.84 ± 1.35	1.66 ± 2.14	0.001
TC (mmol/L)	5.50 ± 2.35	5.14 ± 1.24	5.43 ± 1.39	5.47 ± 1.26	5.50 ± 1.31	5.49 ± 1.15	5.34 ± 1.31	5.39 ± 1.29	5.32 ± 1.18	5.34 ± 1.17	0.576
HDL-C (mmol/L)	1.18 ± 0.32	1.15 ± 0.34	1.19 ± 0.30	1.29 ± 0.37	1.22 ± 0.29	1.21 ± 0.27	1.25 ± 0.33	1.20 ± 0.30	1.26 ± 0.29	1.29 ± 0.32	<0.001
LDL-C (mmol/L)	2.80 ± 1.41	2.68 ± 0.87	2.78 ± 1.04	2.89 ± 0.94	2.95 ± 0.88	2.96 ± 0.88	2.93 ± 1.00	2.89 ± 0.94	2.89 ± 0.91	2.89 ± 0.91	0.438
**Female**											
BMI (kg/㎡)	26.30 ± 4.42	26.02 ± 3.97	25.77 ± 3.77	25.24 ± 3.55	25.82 ± 3.52	25.09 ± 2.91	25.64 ± 3.78	25.44 ± 3.39	24.95 ± 3.32	24.81 ± 2.68	0.020
HBA1c (%)	8.49 ± 2.03	8.47 ± 2.27	8.24 ± 2.02	8.14 ± 2.07	8.29 ± 2.21	8.06 ± 1.99	7.94 ± 2.05	8.09 ± 2.09	7.74 ± 1.91	7.64 ± 1.71	0.035
TG (mmol/L)	2.59 ± 3.33	1.80 ± 1.04	1.82 ± 1.14	1.92 ± 1.74	1.88 ± 1.60	1.72 ± 0.88	1.63 ± 0,89	1.52 ± 0.58	1.61 ± 1.33	1.61 ± 0.80	<0.001
TC (mmol/L)	5.90 ± 1.69	5.84 ± 1.34	5.81 ± 1.37	5.96 ± 1.51	5.71 ± 1.15	5.85 ± 1.23	5.78 ± 1.12	5.79 ± 1.27	6.09 ± 1.33	5.75 ± 1.06	0.633
HDL-C (mmol/L)	1.28 ± 0.33	1.40 ± 0.32	1.36 ± 0.34	1.39 ± 0.33	1.38 ± 0.32	1.35 ± 0.27	1.41 ± 0.29	1.35 ± 0.32	1.50 ± 0.27	1.44 ± 0.34	<0.001
LDL-C (mmol/L)	3.04 ± 1.12	3.14 ± 1.03	3.13 ± 1.06	3.18 ± 1.08	3.05 ± 0.86	3.23 ± 0.98	3.16 ± 0.94	3.26 ± 0.97	3.38 ± 1.03	3.10 ± 0.80	0.287

Among them, one should pay attention to body mass index (BMI), glycated hemoglobin (HbA1c), TG, total cholesterol (TC), low-density lipoprotein cholesterol (LDL-C), and HDL-C. The 25 (OH) D levels were divided into ten equal parts, denoted as 0–9: 0 (≤10.73), 1 (10.74–13.40), 2 (13.41–15.46), 3 (15.47–17.43), 4 (17.44–19.27), 5 (19.28–21.41), 6 (21.42–23.70), 7 (23.71–26.48), 8 (26.49–31.16), and 9 (≥31.17) ng/mL.

### 3.4 Inflammatory markers in 25(OH)D3 and T2DM patients

As shown in Table 3, with the increase of vitamin D, the average levels of blood hsCRP, ESR, white blood cell count, NEUT, and MONO decreased. The trend test was positive (*p* < 0.05), while the average level of peripheral blood LYMPH showed a trend of first increasing and then decreasing. After adjusting for age and gender factors, multiple linear regression analysis showed a negative correlation between ESR, hsCRP, white blood cell count (WBC), and vitamin D levels (*p* < 0.05).

**Table 3:** Different 25(OH)D3 levels and inflammatory markers in diabetic patients

	0	1	2	3	4	5	6	7	8	9	*p*
**Male**											
ESR (mm/h)	28.56 ± 26.79	15.90 ± 22.11	12.40 ± 13.92	10.58 ± 12.60	10.53 ± 10.96	11.33 ± 11.79	10.57 ± 12.87	9.13 ± 8.36	11.18 ± 11.19	10.47 ± 13.18	<0.001
hsCRP (mg/L)	22.45 ± 52.08	10. 15 ± 25.58	6.23 ± 14.16	6.19 ± 25.52	6.69 ± 13.29	4.95 ± 10.72	6.06 ± 19.60	4.69 ± 9.60	5.47 ± 17.70	4.91 ± 15.70	<0.001
WBC (10^9^/L)	7.35 ± 2.31	6.82 ± 2.03	6.41 ± 1.95	6.33 ± 2.90	6.27 ± 1.84	6.39 ± 1.97	6.34 ± 2.10	6.20 ± 1.79	6.14 ± 1.63	6.06 ± 1.82	<0.001
MONO (10^9^/L)	0.54 ± 0.21	0.50 ± 0.15	0.49 ± 0.15	0.46 ± 0.21	0.48 ± 0.17	0.47 ± 0.16	0.46 ± 0.16	0.46 ± 0.16	0.45 ± 0.14	0.46 ± 0.17	0.002
NEUT (10^9^/L)	4.76 ± 2.13	4.09 ± 1.78	3.84 ± 1.64	3.71 ± 2.64	3.70 ± 1.45	3.76 ± 1.63	3.75 ± 1.72	3.66 ± 1.43	3.54 ± 1.32	3.59 ± 1.50	<0.001
LYMPH (10^9^9/L)	1.86 ± 0.77	2.04 ± 0.69	1.88 ± 0.66	1.98 ± 0.66	1.90 ± 0.61	1.98 ± 0.61	1.93 ± 0.57	1.90 ± 0.54	1.95 ± 0.66	1.84 ± 0.58	0.185
**Female**											
ESR (mm/h)	27.22 ± 22.63	20.85 ± 14.91	20.69 ± 16.14	18.39 ± 15.26	17.56 ± 11.74	21.57 ± 18.72	20.34 ± 15.77	19.19 ± 20.80	16.68 ± 15.23	19.11 ± 14.43	<0.001
hsCRP (mg/L)	10.43 ± 21.66	8.30 ± 31.03	6.01 ± 13.79	4.17 ± 4.10	3.61 ± 2.92	5.66 ± 9.81	4.88 ± 8.60	8.56 ± 34.21	4.62 ± 11.32	4.46 ± 9.22	0.028
WBC (10^9^9/L)	6.30 ± 2.19	6.24 ± 1.76	6.01 ± 1.60	5.83 ± 1.56	5.99 ± 1.48	5.88 ± 1.61	5.76 ± 1.55	6.05 ± 2.19	5.79 ± 1.86	5.65 ± 1.72	0.068
MONO (10^9^9/L)	0.44 ± 0.17	0.42 ± 0.17	0.41 ± 0.13	0.40 ± 0.12	0.40 ± 0.13	0.40 ± 0.13	0.38 ± 0.12	0.39 ± 0.15	0.38 ± 0.12	0.37 ± 0.11	<0.001
NEUT (10^9^9/L)	3.85 ± 1.88	3.72 ± 1.47	3.55 ± 1.33	3.40 ± 1.22	3,43 ± 1.07	3.47 ± 1.39	3.29 ± 1.15	3.58 ± 2.03	3.41 ± 1.62	3.35 ± 1.48	0.055
LYMPH (10^9^9/L)	1.88 ± 1.13	1.96 ± 0.71	1.90 ± 0.59	1.89 ± 0.55	2.02 ± 0.65	1.87 ± 0.51	1.94 ± 0.61	1.93 ± 0.54	1.86 ± 0.57	1.81 ± 0.52	0.478

Among them, it should be noted in Table 3 that ESR, hsCRP, WBC, MONO, NEUT, and LYMPH are divided into ten equal parts for 25 (OH) D levels, represented by 0–9, 0 (≤10.73), 1 (10.74–13.40), 2 (13.41–15.46), 3 (15.47–17.43), 4 (17.44–19.27), 5 (19.28 21.41), 6 (21.42 23.70), 7 (23.71 26.48), 8 (26.49 31.16), and 9 (≥31.17) ng/mL.

## 4 Discussion

The results of this study found that vitamin D deficiency/deficiency is prevalent in T2DM patients, with a prevalence rate of 86.78%. The average level of blood 25(OH)D3 is low (males: 22.46 ± 9.53 ng/mL; females: 19.00 ± 8.51 ng/mL). The prevalence of vitamin D deficiency in female patients exceeds 60%, and their 25(OH)D3 deficiency is more severe than in males. The low level of vitamin D is closely related to multiple inflammatory markers.

Previous studies have shown that the vitamin D levels of T2DM patients are lower than those of the normal population. Liu Chuanwei et al. (2019) conducted a study on 796 T2DM patients in Shanghai and found that the prevalence of vitamin D deficiency was 83% [10]. Calvo-Romero et al. conducted a study on 103 patients with T2DM in southern Spain, with a prevalence rate of 69.9% for serum 25(OH)D levels below 20 ng/mL. Females had lower serum 25(OH)D levels than males (13.7 ± 7.3 vs 17.8 ± 9.1 ng/mL), and the proportion of females with serum 25(OH)D levels below 20 ng/mL was significantly higher than males (82.7 vs 56.9%; *p* = 0.002) [11]. These studies are consistent with the results of this study.

The results of this study show a negative correlation between blood 25(OH)D3 levels and HbA1c, which is consistent with some previous research findings. For example, a meta-analysis including 24 clinical trials conducted by Mirhosseini et al. showed that vitamin D supplementation could significantly reduce the levels of FPG and HbA1c in diabetes patients (mean difference: −0.30%; 95% CI: −0.45 to −0.15, *p* < 0.001), (mean difference: −0.27 mmol/L; 95% CI: −1.06 to −0.26, *p* = 0.001) [12]. In 2017, Fu Junling et al. found a negative correlation (*p* = 0.047) between serum 25 hydroxyvitamin D levels and HbA1c (*r* = −0.088) in a study of young people in Beijing [13]. However, in 2018, a foreign meta-analysis reported that vitamin D had no significant effect on glycated hemoglobin [14] (weighted mean difference = −0.11; 95% CI: −0.35–0.13; *p* = 0.38), and was highly heterogeneous (*p* < 0.001; *I* ^2^ = 92%). At present, there is a lack of consensus and more well-designed, longer-duration large-scale studies are needed to further elucidate their impact on baseline HbA1c.

Previous studies have shown a correlation between vitamin D levels and dyslipidemia. Jiang et al. studied the data obtained during routine health checks on 3,788 adults in northern China and found that serum 25(OH)D3 was negatively correlated with LDL-C and TG levels, while positively correlated with HDL-C levels [15]. The research results of Ries et al. also indicate that the level of 25(OH)D3 is negatively correlated with TG and positively correlated with HDL-C. These are consistent with the results of this study [16]. However, Sun et al. found that the serum vitamin D levels in 132 T2DM patients were not related to TG, LDL, and HDL [17]. Therefore, whether there is a correlation between the level of vitamin D and which components in blood lipids are abnormal still needs to be further confirmed by a large number of clinical intervention studies in the future.

Vitamin D deficiency is associated with severe chronic inflammation. Wang Pin et al. (2021) conducted a retrospective analysis of 99 T2DM patients at the People’s Hospital of Lu’an City, and the results indicated that 25(OH)D3 levels in T2DM patients were negatively correlated with neutrophils, white blood cells, and neutrophil/lymphocyte ratios (*r* values were −0.232, −0.201, and −0.317, respectively) [18]. A retrospective analysis by Wang et al. (2021) indicated that in patients with diabetes, those with vitamin D deficiency had the highest NEUT (*p* = 0.001), the lowest LYMPH (*p* = 0.016), and the highest NLR (*p* < 0.001) [19]. A randomized controlled trial conducted by El Hajj et al. in 2020 found that after 6 months of vitamin D supplementation, some inflammatory markers in T2DM patients decreased. Compared to the placebo, the vitamin D group had higher levels of blood 25(OH)D (*p* < 0.0001) and hsCRP, and TNF-α concentration significantly decreased (*p* < 0.0001) [20]. The above research results are consistent with the results of this study, further indicating that 25(OH)D3 in T2DM patients is closely related to chronic low-grade inflammation.

This study is a retrospective investigation and analysis. Due to the limited sample size and geographical location of patients who have been hospitalized in our hospital in recent years, there is a patient selection bias in the research results. As a cross-sectional study, it is not possible to determine whether there is a causal relationship between vitamin D and chronic low-grade inflammation. Therefore, in subsequent studies, it is necessary to conduct long-term follow-up observations on patients with vitamin D deficiency after intervention treatment to clarify the relationship between vitamin D and inflammation.

The “Dietary Guidelines for Chinese Residents” recommends that adults aged 18–64 consume 400 IU (international units) of vitamin D per day, and elderly people over 65 years old consume 600 IU per day. Patients with diabetes often have osteoporosis, and the recommended intake is 800 IU per day. Since vitamin D is a fat-soluble vitamin that cannot be excreted as quickly in urine as water-soluble vitamins, excessive intake may cause cumulative poisoning. Dr. Malachi J. McKenna from University College Dublin and Dr Mary A.T. Flynn of the Irish Food Safety Authority published editorials warning that vitamin D supplementation and vitamin D treatment are not the same. Vitamin D is not the more, the better, and high-dose vitamin D intake is not conducive to good health. The symptoms of excessive vitamin D intake may be manifested as anorexia, weight loss, frequent urination, excessive thirst, irregular heartbeat, etc. In severe cases, it may cause hypercalcemia and hypercalcemia, leading to calcification of blood vessels and damage to the heart, blood vessels, and kidneys.

In summary, vitamin D deficiency is very common in T2DM patients, and inflammatory reactions in T2DM may be related to a decrease in 25(OH)D3 levels in the blood. Clinical work should pay attention to testing and supplementing vitamin D in T2DM patients appropriately. However, further research is needed to clarify whether supplementing vitamin D can serve as an adjuvant treatment for preventing chronic inflammation in T2DM patients.
